# A triclinic polymorph of *N*-[4-(4-methyl­benzene­sulfonamido)­phenyl­sulfon­yl]acetamide

**DOI:** 10.1107/S1600536812008616

**Published:** 2012-03-07

**Authors:** Khizar Hayat, Muhammad Nadeem Asghar, M. Nawaz Tahir, Muhammad Shafiq, Dildar Ahmad

**Affiliations:** a Forman Christian College (A Chartered University), Ferozepur Road, Lahore 54600, Pakistan; b Department of Physics, University of Sargodha, Sargodha, Pakistan; c Department of Chemistry, GC University, Lahore 54000, Pakistan

## Abstract

In the asymmetric unit of the title compound, C_15_H_16_N_2_O_5_S_2_, there are two symmetry-independent mol­ecules which adopt similar conformations, with dihedral angles between the aromatic rings of 59.30 (8) and 61.81 (8)°, and dihedral angles between acetamide group and the benzene ring of 77.08 (10) and 78.40 (10)°. Each type of mol­ecule forms similar one-dimensional polymeric structures extending along the *b* axis *via* N—H⋯O hydrogen bonds. These hydrogen bonds generate two types of centrosymmetric motifs, *R*_2_^2^(8) and *R*_2_^2^(20). Moreover C—H⋯O inter­actions assemble the mol­ecules into a three-dimensional framework. The crystal structure was determined from a non-merohedral twin [ratio of the twin components = 0.322 (4):0.678 (4)].

## Related literature

For a monoclinic polymorph of the title compound, see: Ashfaq *et al.* (2010 ▶). For graph-set notation, see: Bernstein *et al.* (1995 ▶).
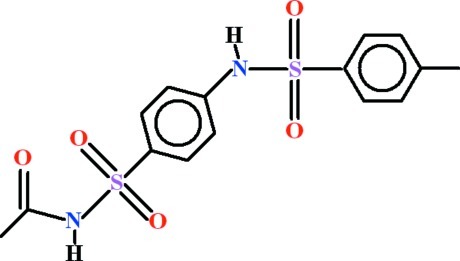


## Experimental

### 

#### Crystal data

C_15_H_16_N_2_O_5_S_2_
*M* *_r_* = 368.42
Triclinic, 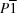

*a* = 9.6722 (3) Å
*b* = 11.9968 (4) Å
*c* = 15.4784 (6) Å
α = 82.802 (2)°
β = 79.232 (1)°
γ = 89.653 (2)°
*V* = 1750.24 (11) Å^3^
*Z* = 4
Mo *K*α radiation
μ = 0.33 mm^−1^
*T* = 296 K
0.35 × 0.25 × 0.22 mm

#### Data collection

Bruker Kappa APEXII CCD diffractometer
Absorption correction: multi-scan (*SADABS*; Bruker, 2005 ▶) *T*_min_ = 0.915, *T*_max_ = 0.938
31011 measured reflections
8382 independent reflections
5598 reflections with *I* > 2σ(*I*)
*R*_int_ = 0.028

#### Refinement

*R*[*F* ^2^ > 2σ(*F* ^2^)] = 0.053
*wR*(*F* ^2^) = 0.153
*S* = 1.02
8382 reflections
438 parameters
H-atom parameters constrained
Δρ_max_ = 0.52 e Å^−3^
Δρ_min_ = −0.45 e Å^−3^

### 

Data collection: *APEX2* (Bruker, 2009 ▶); cell refinement: *SAINT* (Bruker, 2009 ▶); data reduction: *SAINT*; program(s) used to solve structure: *SHELXS97* (Sheldrick, 2008 ▶); program(s) used to refine structure: *SHELXL97* (Sheldrick, 2008 ▶); molecular graphics: *ORTEP-3 for Windows* (Farrugia, 1997 ▶) and *PLATON* (Spek, 2009 ▶); software used to prepare material for publication: *WinGX* (Farrugia, 1999 ▶) and *PLATON*.

## Supplementary Material

Crystal structure: contains datablock(s) global, I. DOI: 10.1107/S1600536812008616/gk2456sup1.cif

Structure factors: contains datablock(s) I. DOI: 10.1107/S1600536812008616/gk2456Isup2.hkl

Supplementary material file. DOI: 10.1107/S1600536812008616/gk2456Isup3.cml

Additional supplementary materials:  crystallographic information; 3D view; checkCIF report

## Figures and Tables

**Table 1:** Hydrogen-bond geometry (Å, °)

*D*—H⋯*A*	*D*—H	H⋯*A*	*D*⋯*A*	*D*—H⋯*A*
N1—H1⋯O5^i^	0.86	2.14	2.841 (3)	138
N2—H2*A*⋯O4^ii^	0.86	2.06	2.876 (3)	158
N3—H3*A*⋯O10^iii^	0.86	2.18	2.859 (3)	136
N4—H4⋯O8^iv^	0.86	2.05	2.867 (3)	157
C13—H13⋯O2	0.93	2.49	3.062 (4)	120
C22—H22*A*⋯O6^v^	0.96	2.59	3.338 (5)	135
C24—H24⋯O6	0.93	2.46	3.026 (4)	119

Symmetry codes: (i) 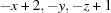; (ii) 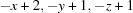; (iii) 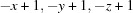; (iv) 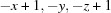; (v) 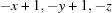.

**Fig. 1.**
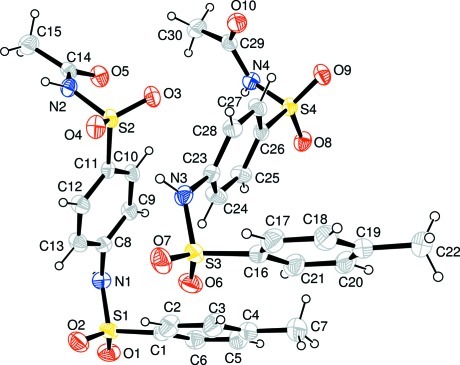
View of the title compound with the displacement ellipsoids drawn at the 30% probability level. H-atoms are shown by small circles of arbitrary radii.

**Fig. 2.**
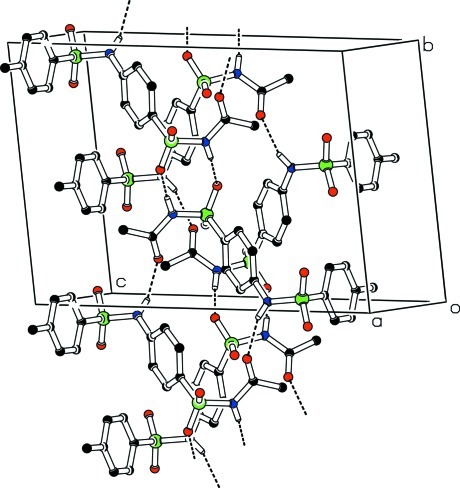
The partial packing (PLATON; Spek, 2009) which shows that molecules form dimers forming R22(8) ring motif and dimers are interlinked and complete R22(20) ring motif. The H-atoms not involved in hydrogen bonding have been omitted for clarity.

## supplementary crystallographic information

### Comment

The title compound (I), (Fig. 1) has been synthesized as a part of the series of sulfonamide derivatives. The aim of our research work is to find the potential sulfonamide derivatives possesing anti-microbial activity. The crystal structure of the monoclinic polymorph of the title compound has been reported earlier by Ashfaq *et al.* (2010). The molecules in the two polymorphs differ in conformation.

In (I), two molecules in the asymmetric unit are present, which differ from each other geometrically. In one molecule, the toluene group A (C1–C7), benzene ring B (C8–C13) and the acetamide group C (N2/C14/C15/O5) are planar with r. m. s. deviation of 0.0089 Å, 0.0080 Å and 0.0028 Å, respectively. The dihedral angles between A/B, A/C and B/C are 61.81 (8)°, 45.90 (14)° and 77.08 (10)°, respectively. In second molecule, the toluene group D (C16–C22), benzene ring E (C23–C28) and the acetamide group F (N4/C29/C30/O10) are planar with r. m. s. deviation of 0.0116 Å, 0.0066 Å and 0.0006 Å, respectively. The dihedral angles between D/E, D/F and E/F are 59.30 (8)°, 46.10 (14)° and 78.40 (10)°, respectively. The dihedral angle between two aromatic rings in its polymrph (Ashfaq *et al.*, 2010) is 81.33 (6)° compared to 61.81 (8)° and 59.30 (8)°. In both molecules, there exist weak intramolecular H–bonding of C—H···O type (Table 1). Both molecules are dimerized themselves due to intermolecular H-bonding of N—H···O type forming *R*_2_^2^(8) ring motifs (Bernstein *et al.*, 1995). The dimers are interliked due to strong N—H···O type of H–bondings (Table 1, Fig. 2) and form *R*_2_^2^(20) ring motifs.

### Experimental

Equal molar ratio of *N*-[(4-aminophenyl)sulfonyl]acetamide and *p*-toluene sulfonyl chloride was dissolved in 20 ml distilled water. The solution with pH = 8–9 adjusted using Na_2_CO_3_ (1 *M*) was stirrered at room temperature for 6 h. Progress of the reaction was monitored by the consumption of suspended *p*-toluene sulfonyl chloride. On completion, pH was adjusted to 2–3 using HCl (2 N). The precipitate formed was filtered, washed with ditilled water and recrystallized from methanol to afford colorless prisms with m.p. 385 K.

### Refinement

The crystal structure was solved from non-merohedral twin with the twin law in the reciprocal space of 0.211, 0.700, 280.000: 1.211, 0.211, 0.000: 0.429, 0.429, 1.000 and the twin component ratio of 0.322 (4)/0.678 (4). In the refinement the HKLF 5 reflection file format in *SHELXL* was used.

The H-atoms were positioned geometrically (N—H = 0.86 Å, C—H = 0.93–0.97 Å) and refined as riding with *U*_iso_(H) = *xU*_eq_(C, N), where *x* = 1.5 for methyl groups and *x* = 1.2 for all other H-atoms.

### Crystal data

**Table:**

C_15_H_16_N_2_O_5_S_2_	*Z* = 4
*M**_r_* = 368.42	*F*(000) = 768
Triclinic, *P*1	*D*_x_ = 1.398 Mg m^−^^3^
Hall symbol: -P 1	Mo *K*α radiation, λ = 0.71073 Å
*a* = 9.6722 (3) Å	Cell parameters from 4587 reflections
*b* = 11.9968 (4) Å	θ = 1.7–25.0°
*c* = 15.4784 (6) Å	µ = 0.33 mm^−^^1^
α = 82.802 (2)°	*T* = 296 K
β = 79.232 (1)°	Prism, colorless
γ = 89.653 (2)°	0.35 × 0.25 × 0.22 mm
*V* = 1750.24 (11) Å^3^	

### Data collection

**Table:**

Bruker Kappa APEXII CCD diffractometer	8382 independent reflections
Radiation source: fine-focus sealed tube	5598 reflections with *I* > 2σ(*I*)
Graphite monochromator	*R*_int_ = 0.028
Detector resolution: 7.60 pixels mm^-1^	θ_max_ = 28.3°, θ_min_ = 1.7°
ω scans	*h* = −12→12
Absorption correction: multi-scan (*SADABS*; Bruker, 2005)	*k* = −15→15
*T*_min_ = 0.915, *T*_max_ = 0.938	*l* = −20→20
31011 measured reflections	

### Refinement

**Table:**

Refinement on *F*^2^	Primary atom site location: structure-invariant direct methods
Least-squares matrix: full	Secondary atom site location: difference Fourier map
*R*[*F*^2^ > 2σ(*F*^2^)] = 0.053	Hydrogen site location: inferred from neighbouring sites
*wR*(*F*^2^) = 0.153	H-atom parameters constrained
*S* = 1.02	*w* = 1/[σ^2^(*F*_o_^2^) + (0.0652*P*)^2^ + 0.7795*P*] where *P* = (*F*_o_^2^ + 2*F*_c_^2^)/3
8382 reflections	(Δ/σ)_max_ = 0.001
438 parameters	Δρ_max_ = 0.52 e Å^−^^3^
0 restraints	Δρ_min_ = −0.45 e Å^−^^3^

### Special details

**Table:**

Geometry. Bond distances, angles *etc*. have been calculated using the rounded fractional coordinates. All su's are estimated from the variances of the (full) variance-covariance matrix. The cell e.s.d.'s are taken into account in the estimation of distances, angles and torsion angles
Refinement. Refinement of *F*^2^ against ALL reflections. The weighted *R*-factor *wR* and goodness of fit *S* are based on *F*^2^, conventional *R*-factors *R* are based on *F*, with *F* set to zero for negative *F*^2^. The threshold expression of *F*^2^ > σ(*F*^2^) is used only for calculating *R*-factors(gt) *etc*. and is not relevant to the choice of reflections for refinement. *R*-factors based on *F*^2^ are statistically about twice as large as those based on *F*, and *R*- factors based on ALL data will be even larger.

### Fractional atomic coordinates and isotropic or equivalent isotropic displacement parameters (Å^2^)

**Table:**

	*x*	*y*	*z*	*U*_iso_*/*U*_eq_	
S1	1.18463 (8)	0.05657 (7)	0.15628 (5)	0.0535 (3)	
S2	0.84421 (7)	0.35232 (5)	0.49521 (5)	0.0415 (2)	
O1	1.2489 (3)	−0.0454 (2)	0.13292 (16)	0.0730 (9)	
O2	1.2545 (2)	0.1621 (2)	0.12469 (15)	0.0698 (8)	
O3	0.70702 (19)	0.30834 (16)	0.53244 (14)	0.0515 (7)	
O4	0.8603 (2)	0.46483 (15)	0.44956 (14)	0.0523 (7)	
O5	0.8812 (2)	0.18161 (16)	0.64458 (14)	0.0585 (7)	
N1	1.1577 (3)	0.0404 (2)	0.26522 (15)	0.0494 (8)	
N2	0.9331 (2)	0.35691 (18)	0.57571 (15)	0.0441 (7)	
C1	1.0166 (3)	0.0638 (3)	0.12810 (18)	0.0500 (9)	
C2	0.9520 (4)	0.1664 (3)	0.1181 (2)	0.0687 (12)	
C3	0.8155 (4)	0.1685 (4)	0.1015 (3)	0.0785 (16)	
C4	0.7443 (4)	0.0727 (4)	0.0942 (2)	0.0710 (14)	
C5	0.8117 (4)	−0.0286 (4)	0.1023 (2)	0.0747 (14)	
C6	0.9484 (4)	−0.0339 (3)	0.1193 (2)	0.0650 (12)	
C7	0.5962 (4)	0.0779 (5)	0.0770 (3)	0.107 (2)	
C8	1.0824 (3)	0.1175 (2)	0.31772 (17)	0.0423 (8)	
C9	0.9745 (3)	0.0768 (2)	0.38598 (18)	0.0464 (9)	
C10	0.9013 (3)	0.1481 (2)	0.44060 (19)	0.0450 (8)	
C11	0.9352 (3)	0.2614 (2)	0.42570 (17)	0.0380 (8)	
C12	1.0441 (3)	0.3032 (2)	0.35796 (19)	0.0516 (10)	
C13	1.1186 (3)	0.2313 (2)	0.3047 (2)	0.0562 (10)	
C14	0.9428 (3)	0.2699 (2)	0.64153 (18)	0.0447 (9)	
C15	1.0310 (4)	0.2954 (3)	0.7061 (2)	0.0662 (12)	
S3	0.69039 (8)	0.54642 (6)	0.15666 (5)	0.0506 (2)	
S4	0.34505 (7)	0.15067 (5)	0.49579 (5)	0.0415 (2)	
O6	0.7593 (2)	0.4488 (2)	0.12796 (15)	0.0663 (8)	
O7	0.7553 (2)	0.65458 (19)	0.12984 (16)	0.0685 (8)	
O8	0.3612 (2)	0.05275 (15)	0.45035 (14)	0.0517 (7)	
O9	0.20777 (19)	0.18511 (16)	0.53232 (14)	0.0522 (7)	
O10	0.3820 (2)	0.27238 (17)	0.64627 (14)	0.0581 (7)	
N3	0.6647 (3)	0.53153 (19)	0.26533 (15)	0.0473 (8)	
N4	0.4330 (2)	0.11853 (18)	0.57696 (15)	0.0435 (7)	
C16	0.5211 (3)	0.5480 (3)	0.13051 (18)	0.0494 (9)	
C17	0.4500 (4)	0.6488 (3)	0.1236 (2)	0.0664 (12)	
C18	0.3142 (4)	0.6490 (4)	0.1090 (3)	0.0805 (16)	
C19	0.2471 (4)	0.5512 (4)	0.1003 (2)	0.0774 (16)	
C20	0.3210 (4)	0.4524 (4)	0.1049 (2)	0.0762 (14)	
C21	0.4574 (4)	0.4491 (3)	0.1199 (2)	0.0630 (11)	
C22	0.0974 (4)	0.5515 (6)	0.0856 (3)	0.121 (3)	
C23	0.5887 (3)	0.4391 (2)	0.31887 (17)	0.0412 (8)	
C24	0.6247 (3)	0.3287 (2)	0.3074 (2)	0.0533 (10)	
C25	0.5486 (3)	0.2412 (2)	0.36048 (19)	0.0506 (9)	
C26	0.4380 (3)	0.2630 (2)	0.42648 (17)	0.0389 (8)	
C27	0.4030 (3)	0.3724 (2)	0.43997 (19)	0.0457 (8)	
C28	0.4781 (3)	0.4596 (2)	0.38568 (18)	0.0452 (8)	
C29	0.4428 (3)	0.1842 (2)	0.64282 (18)	0.0450 (9)	
C30	0.5321 (4)	0.1372 (3)	0.7070 (2)	0.0655 (11)	
H1	1.19113	−0.01807	0.29175	0.0592*	
H2	0.99857	0.23250	0.12244	0.0824*	
H2A	0.97704	0.41865	0.57684	0.0530*	
H3	0.77109	0.23718	0.09504	0.0937*	
H5	0.76554	−0.09420	0.09641	0.0899*	
H6	0.99324	−0.10250	0.12462	0.0780*	
H7A	0.58829	0.14113	0.03363	0.1594*	
H7B	0.53193	0.08598	0.13109	0.1594*	
H7C	0.57375	0.01005	0.05524	0.1594*	
H9	0.95104	0.00057	0.39512	0.0557*	
H10	0.82975	0.12018	0.48707	0.0540*	
H12	1.06666	0.37953	0.34857	0.0619*	
H13	1.19302	0.25870	0.26004	0.0675*	
H15A	1.08342	0.23020	0.72170	0.0989*	
H15B	0.97142	0.31599	0.75833	0.0989*	
H15C	1.09487	0.35648	0.67974	0.0989*	
H3A	0.69879	0.58185	0.29084	0.0568*	
H4	0.47630	0.05569	0.57835	0.0522*	
H17	0.49406	0.71564	0.12870	0.0796*	
H18	0.26661	0.71646	0.10498	0.0965*	
H20	0.27760	0.38609	0.09762	0.0914*	
H21	0.50538	0.38172	0.12281	0.0753*	
H22A	0.09375	0.52163	0.03134	0.1804*	
H22B	0.06371	0.62703	0.08201	0.1804*	
H22C	0.03928	0.50590	0.13413	0.1804*	
H24	0.70038	0.31417	0.26384	0.0640*	
H25	0.57139	0.16744	0.35203	0.0608*	
H27	0.32961	0.38673	0.48515	0.0548*	
H28	0.45444	0.53331	0.39387	0.0542*	
H30A	0.47464	0.09206	0.75606	0.0981*	
H30B	0.57526	0.19760	0.72820	0.0981*	
H30C	0.60386	0.09164	0.67805	0.0981*	

### Atomic displacement parameters (Å^2^)

**Table:**

	*U*^11^	*U*^22^	*U*^33^	*U*^12^	*U*^13^	*U*^23^
S1	0.0472 (4)	0.0611 (5)	0.0493 (4)	0.0080 (3)	−0.0011 (3)	−0.0085 (3)
S2	0.0363 (3)	0.0328 (3)	0.0565 (4)	0.0002 (3)	−0.0108 (3)	−0.0063 (3)
O1	0.0676 (15)	0.0839 (17)	0.0679 (15)	0.0294 (13)	−0.0038 (12)	−0.0259 (12)
O2	0.0589 (14)	0.0778 (16)	0.0642 (14)	−0.0104 (12)	0.0028 (11)	0.0026 (12)
O3	0.0348 (10)	0.0470 (11)	0.0718 (13)	−0.0029 (8)	−0.0058 (9)	−0.0101 (9)
O4	0.0540 (12)	0.0328 (10)	0.0723 (13)	0.0028 (8)	−0.0208 (10)	−0.0016 (9)
O5	0.0577 (13)	0.0408 (11)	0.0734 (14)	−0.0052 (10)	−0.0097 (10)	0.0028 (10)
N1	0.0536 (14)	0.0459 (13)	0.0485 (13)	0.0113 (11)	−0.0110 (11)	−0.0038 (10)
N2	0.0470 (13)	0.0358 (11)	0.0512 (13)	−0.0061 (10)	−0.0130 (10)	−0.0056 (10)
C1	0.0504 (16)	0.0572 (18)	0.0418 (15)	0.0054 (14)	−0.0056 (12)	−0.0088 (13)
C2	0.073 (2)	0.063 (2)	0.078 (2)	0.0111 (18)	−0.0283 (19)	−0.0188 (17)
C3	0.075 (2)	0.085 (3)	0.086 (3)	0.030 (2)	−0.033 (2)	−0.026 (2)
C4	0.057 (2)	0.105 (3)	0.0506 (19)	0.001 (2)	−0.0073 (15)	−0.0122 (19)
C5	0.073 (2)	0.085 (3)	0.065 (2)	−0.024 (2)	−0.0119 (18)	−0.0052 (19)
C6	0.074 (2)	0.061 (2)	0.058 (2)	0.0000 (18)	−0.0095 (17)	−0.0041 (15)
C7	0.064 (3)	0.174 (5)	0.083 (3)	−0.001 (3)	−0.019 (2)	−0.014 (3)
C8	0.0442 (14)	0.0397 (14)	0.0443 (14)	0.0046 (11)	−0.0120 (11)	−0.0054 (11)
C9	0.0491 (16)	0.0327 (13)	0.0562 (17)	−0.0046 (12)	−0.0066 (13)	−0.0058 (12)
C10	0.0404 (14)	0.0388 (14)	0.0536 (16)	−0.0057 (11)	−0.0042 (12)	−0.0036 (12)
C11	0.0360 (13)	0.0313 (12)	0.0484 (15)	−0.0019 (10)	−0.0116 (11)	−0.0057 (10)
C12	0.0583 (18)	0.0345 (14)	0.0574 (18)	−0.0089 (13)	−0.0008 (14)	−0.0027 (12)
C13	0.0577 (18)	0.0446 (16)	0.0587 (18)	−0.0117 (14)	0.0060 (14)	−0.0016 (13)
C14	0.0380 (14)	0.0435 (15)	0.0500 (16)	0.0057 (12)	−0.0003 (12)	−0.0076 (12)
C15	0.067 (2)	0.075 (2)	0.059 (2)	−0.0006 (18)	−0.0183 (16)	−0.0083 (16)
S3	0.0428 (4)	0.0556 (4)	0.0502 (4)	−0.0075 (3)	−0.0006 (3)	−0.0061 (3)
S4	0.0370 (3)	0.0335 (3)	0.0558 (4)	0.0017 (3)	−0.0102 (3)	−0.0105 (3)
O6	0.0583 (13)	0.0745 (15)	0.0614 (14)	0.0071 (11)	0.0061 (10)	−0.0172 (11)
O7	0.0567 (13)	0.0655 (14)	0.0760 (15)	−0.0230 (11)	−0.0049 (11)	0.0085 (11)
O8	0.0558 (12)	0.0375 (10)	0.0670 (13)	0.0009 (9)	−0.0179 (10)	−0.0167 (9)
O9	0.0357 (10)	0.0473 (11)	0.0733 (14)	0.0022 (8)	−0.0071 (9)	−0.0108 (9)
O10	0.0561 (12)	0.0486 (12)	0.0730 (14)	0.0048 (10)	−0.0105 (10)	−0.0238 (10)
N3	0.0508 (14)	0.0434 (12)	0.0499 (13)	−0.0085 (10)	−0.0123 (10)	−0.0094 (10)
N4	0.0463 (12)	0.0333 (11)	0.0522 (13)	0.0067 (10)	−0.0115 (10)	−0.0075 (9)
C16	0.0480 (16)	0.0589 (18)	0.0405 (15)	−0.0075 (14)	−0.0046 (12)	−0.0086 (12)
C17	0.065 (2)	0.067 (2)	0.073 (2)	0.0022 (18)	−0.0188 (17)	−0.0230 (17)
C18	0.064 (2)	0.106 (3)	0.080 (3)	0.019 (2)	−0.0204 (19)	−0.035 (2)
C19	0.0507 (19)	0.136 (4)	0.0482 (19)	−0.011 (2)	−0.0060 (15)	−0.026 (2)
C20	0.074 (2)	0.098 (3)	0.056 (2)	−0.042 (2)	−0.0102 (18)	−0.0086 (19)
C21	0.069 (2)	0.065 (2)	0.0556 (19)	−0.0159 (17)	−0.0133 (16)	−0.0063 (15)
C22	0.057 (2)	0.232 (7)	0.084 (3)	−0.010 (3)	−0.017 (2)	−0.058 (4)
C23	0.0421 (14)	0.0379 (13)	0.0457 (15)	0.0003 (11)	−0.0125 (11)	−0.0075 (11)
C24	0.0525 (17)	0.0449 (16)	0.0581 (18)	0.0098 (13)	0.0056 (14)	−0.0136 (13)
C25	0.0606 (18)	0.0347 (14)	0.0553 (17)	0.0108 (13)	−0.0033 (14)	−0.0128 (12)
C26	0.0360 (13)	0.0343 (13)	0.0486 (15)	0.0025 (10)	−0.0110 (11)	−0.0093 (11)
C27	0.0389 (14)	0.0419 (14)	0.0550 (16)	0.0079 (12)	−0.0007 (12)	−0.0139 (12)
C28	0.0471 (15)	0.0337 (13)	0.0550 (16)	0.0065 (11)	−0.0063 (12)	−0.0121 (11)
C29	0.0403 (14)	0.0422 (15)	0.0499 (16)	−0.0041 (12)	−0.0003 (12)	−0.0084 (12)
C30	0.067 (2)	0.078 (2)	0.0555 (19)	0.0052 (18)	−0.0188 (16)	−0.0127 (16)

### Geometric parameters (Å, º)

**Table:**

S1—O1	1.425 (3)	C3—H3	0.9300
S1—O2	1.425 (2)	C5—H5	0.9300
S1—N1	1.644 (2)	C6—H6	0.9300
S1—C1	1.759 (3)	C7—H7A	0.9600
S2—O3	1.421 (2)	C7—H7B	0.9600
S2—O4	1.438 (2)	C7—H7C	0.9600
S2—N2	1.647 (2)	C9—H9	0.9300
S2—C11	1.747 (3)	C10—H10	0.9300
S3—O7	1.424 (2)	C12—H12	0.9300
S3—N3	1.641 (2)	C13—H13	0.9300
S3—O6	1.423 (2)	C15—H15C	0.9600
S3—C16	1.758 (3)	C15—H15B	0.9600
S4—O9	1.423 (2)	C15—H15A	0.9600
S4—N4	1.651 (2)	C16—C17	1.390 (5)
S4—O8	1.436 (2)	C16—C21	1.382 (5)
S4—C26	1.751 (3)	C17—C18	1.374 (6)
O5—C14	1.210 (3)	C18—C19	1.378 (7)
O10—C29	1.209 (3)	C19—C20	1.382 (6)
N1—C8	1.416 (4)	C19—C22	1.507 (6)
N2—C14	1.380 (3)	C20—C21	1.381 (6)
N1—H1	0.8600	C23—C28	1.386 (4)
N2—H2A	0.8600	C23—C24	1.392 (3)
N3—C23	1.417 (4)	C24—C25	1.373 (4)
N4—C29	1.381 (3)	C25—C26	1.383 (4)
N3—H3A	0.8600	C26—C27	1.385 (3)
N4—H4	0.8600	C27—C28	1.374 (4)
C1—C6	1.382 (5)	C29—C30	1.492 (5)
C1—C2	1.382 (5)	C17—H17	0.9300
C2—C3	1.391 (6)	C18—H18	0.9300
C3—C4	1.371 (6)	C20—H20	0.9300
C4—C5	1.379 (7)	C21—H21	0.9300
C4—C7	1.505 (6)	C22—H22A	0.9600
C5—C6	1.395 (5)	C22—H22B	0.9600
C8—C13	1.392 (3)	C22—H22C	0.9600
C8—C9	1.382 (4)	C24—H24	0.9300
C9—C10	1.378 (4)	C25—H25	0.9300
C10—C11	1.381 (3)	C27—H27	0.9300
C11—C12	1.387 (4)	C28—H28	0.9300
C12—C13	1.375 (4)	C30—H30A	0.9600
C14—C15	1.490 (5)	C30—H30B	0.9600
C2—H2	0.9300	C30—H30C	0.9600
			
O1—S1—O2	120.70 (15)	C4—C7—H7B	110.00
O1—S1—N1	104.06 (14)	C4—C7—H7C	110.00
O1—S1—C1	109.20 (17)	C4—C7—H7A	109.00
O2—S1—N1	108.23 (14)	H7B—C7—H7C	109.00
O2—S1—C1	107.86 (15)	C8—C9—H9	120.00
N1—S1—C1	105.84 (14)	C10—C9—H9	120.00
O3—S2—O4	119.35 (12)	C11—C10—H10	120.00
O3—S2—N2	108.69 (12)	C9—C10—H10	120.00
O3—S2—C11	110.10 (13)	C13—C12—H12	120.00
O4—S2—N2	103.10 (12)	C11—C12—H12	120.00
O4—S2—C11	108.62 (12)	C8—C13—H13	120.00
N2—S2—C11	106.04 (12)	C12—C13—H13	120.00
N3—S3—C16	105.24 (14)	C14—C15—H15C	109.00
O7—S3—C16	109.50 (15)	H15B—C15—H15C	110.00
O6—S3—N3	107.70 (13)	C14—C15—H15A	109.00
O6—S3—C16	108.13 (15)	H15A—C15—H15B	110.00
O6—S3—O7	120.60 (14)	H15A—C15—H15C	110.00
O7—S3—N3	104.61 (14)	C14—C15—H15B	109.00
O8—S4—C26	108.63 (12)	S3—C16—C17	119.5 (3)
O9—S4—N4	108.81 (12)	S3—C16—C21	120.2 (3)
O8—S4—N4	102.93 (12)	C17—C16—C21	120.3 (3)
O8—S4—O9	119.59 (12)	C16—C17—C18	119.6 (4)
O9—S4—C26	109.97 (13)	C17—C18—C19	121.3 (4)
N4—S4—C26	105.93 (12)	C18—C19—C22	121.2 (5)
S1—N1—C8	123.5 (2)	C20—C19—C22	120.6 (5)
S2—N2—C14	125.40 (18)	C18—C19—C20	118.2 (4)
C8—N1—H1	118.00	C19—C20—C21	122.0 (4)
S1—N1—H1	118.00	C16—C21—C20	118.6 (4)
S2—N2—H2A	117.00	C24—C23—C28	119.4 (2)
C14—N2—H2A	117.00	N3—C23—C24	121.6 (2)
S3—N3—C23	122.68 (19)	N3—C23—C28	119.0 (2)
S4—N4—C29	125.40 (18)	C23—C24—C25	120.0 (3)
C23—N3—H3A	119.00	C24—C25—C26	119.9 (2)
S3—N3—H3A	119.00	S4—C26—C25	119.41 (19)
C29—N4—H4	117.00	S4—C26—C27	119.8 (2)
S4—N4—H4	117.00	C25—C26—C27	120.7 (2)
C2—C1—C6	120.6 (3)	C26—C27—C28	119.1 (3)
S1—C1—C6	119.6 (3)	C23—C28—C27	120.8 (2)
S1—C1—C2	119.8 (3)	O10—C29—C30	124.3 (3)
C1—C2—C3	118.4 (4)	N4—C29—C30	114.7 (2)
C2—C3—C4	122.2 (4)	O10—C29—N4	121.0 (3)
C3—C4—C5	118.6 (4)	C16—C17—H17	120.00
C5—C4—C7	120.6 (4)	C18—C17—H17	120.00
C3—C4—C7	120.8 (4)	C17—C18—H18	119.00
C4—C5—C6	120.8 (4)	C19—C18—H18	119.00
C1—C6—C5	119.4 (4)	C19—C20—H20	119.00
C9—C8—C13	119.7 (2)	C21—C20—H20	119.00
N1—C8—C13	121.7 (3)	C16—C21—H21	121.00
N1—C8—C9	118.5 (2)	C20—C21—H21	121.00
C8—C9—C10	120.6 (2)	C19—C22—H22A	109.00
C9—C10—C11	119.4 (3)	C19—C22—H22B	110.00
S2—C11—C12	119.58 (19)	C19—C22—H22C	109.00
S2—C11—C10	119.7 (2)	H22A—C22—H22B	109.00
C10—C11—C12	120.6 (2)	H22A—C22—H22C	109.00
C11—C12—C13	119.7 (2)	H22B—C22—H22C	109.00
C8—C13—C12	119.9 (3)	C23—C24—H24	120.00
N2—C14—C15	114.8 (2)	C25—C24—H24	120.00
O5—C14—C15	124.7 (3)	C24—C25—H25	120.00
O5—C14—N2	120.5 (3)	C26—C25—H25	120.00
C1—C2—H2	121.00	C26—C27—H27	120.00
C3—C2—H2	121.00	C28—C27—H27	120.00
C4—C3—H3	119.00	C23—C28—H28	120.00
C2—C3—H3	119.00	C27—C28—H28	120.00
C4—C5—H5	120.00	C29—C30—H30A	109.00
C6—C5—H5	120.00	C29—C30—H30B	109.00
C1—C6—H6	120.00	C29—C30—H30C	109.00
C5—C6—H6	120.00	H30A—C30—H30B	109.00
H7A—C7—H7C	109.00	H30A—C30—H30C	109.00
H7A—C7—H7B	109.00	H30B—C30—H30C	109.00
			
O1—S1—N1—C8	−173.5 (3)	S4—N4—C29—O10	1.7 (4)
O2—S1—N1—C8	57.0 (3)	S4—N4—C29—C30	−178.4 (2)
C1—S1—N1—C8	−58.4 (3)	C2—C1—C6—C5	−1.6 (4)
O1—S1—C1—C2	−159.9 (2)	S1—C1—C2—C3	−175.9 (3)
O1—S1—C1—C6	22.5 (3)	C6—C1—C2—C3	1.8 (5)
O2—S1—C1—C2	−27.0 (3)	S1—C1—C6—C5	176.1 (2)
O2—S1—C1—C6	155.3 (2)	C1—C2—C3—C4	−0.5 (6)
N1—S1—C1—C2	88.7 (3)	C2—C3—C4—C5	−1.0 (6)
N1—S1—C1—C6	−89.0 (3)	C2—C3—C4—C7	179.5 (4)
O3—S2—N2—C14	48.6 (2)	C7—C4—C5—C6	−179.2 (3)
O4—S2—N2—C14	176.2 (2)	C3—C4—C5—C6	1.3 (5)
C11—S2—N2—C14	−69.7 (2)	C4—C5—C6—C1	0.0 (5)
O3—S2—C11—C10	−27.0 (3)	N1—C8—C13—C12	−179.1 (3)
O3—S2—C11—C12	155.3 (2)	C13—C8—C9—C10	0.8 (4)
O4—S2—C11—C10	−159.3 (2)	N1—C8—C9—C10	177.9 (3)
O4—S2—C11—C12	22.9 (3)	C9—C8—C13—C12	−2.0 (4)
N2—S2—C11—C10	90.4 (3)	C8—C9—C10—C11	1.0 (4)
N2—S2—C11—C12	−87.3 (2)	C9—C10—C11—S2	−179.4 (2)
N3—S3—C16—C17	86.6 (3)	C9—C10—C11—C12	−1.6 (4)
N3—S3—C16—C21	−91.3 (3)	S2—C11—C12—C13	178.2 (2)
O6—S3—N3—C23	−57.2 (3)	C10—C11—C12—C13	0.4 (4)
O7—S3—N3—C23	173.4 (2)	C11—C12—C13—C8	1.4 (4)
C16—S3—N3—C23	58.0 (3)	S3—C16—C17—C18	−175.7 (3)
O6—S3—C16—C17	−158.6 (2)	C21—C16—C17—C18	2.1 (5)
O6—S3—C16—C21	23.6 (3)	S3—C16—C21—C20	176.0 (2)
O7—S3—C16—C17	−25.4 (3)	C17—C16—C21—C20	−1.8 (4)
O7—S3—C16—C21	156.8 (2)	C16—C17—C18—C19	−0.5 (6)
O8—S4—N4—C29	−176.4 (2)	C17—C18—C19—C20	−1.3 (6)
O9—S4—N4—C29	−48.6 (2)	C17—C18—C19—C22	179.1 (4)
C26—S4—N4—C29	69.6 (2)	C18—C19—C20—C21	1.6 (5)
O8—S4—C26—C25	−23.8 (3)	C22—C19—C20—C21	−178.8 (3)
O8—S4—C26—C27	158.2 (2)	C19—C20—C21—C16	0.0 (5)
O9—S4—C26—C25	−156.4 (2)	N3—C23—C24—C25	−180.0 (3)
O9—S4—C26—C27	25.6 (3)	C28—C23—C24—C25	1.7 (4)
N4—S4—C26—C25	86.2 (2)	N3—C23—C28—C27	−179.0 (3)
N4—S4—C26—C27	−91.9 (2)	C24—C23—C28—C27	−0.6 (4)
S1—N1—C8—C9	128.8 (3)	C23—C24—C25—C26	−1.3 (4)
S1—N1—C8—C13	−54.2 (4)	C24—C25—C26—S4	−178.2 (2)
S2—N2—C14—C15	179.5 (2)	C24—C25—C26—C27	−0.2 (4)
S2—N2—C14—O5	−1.4 (4)	S4—C26—C27—C28	179.2 (2)
S3—N3—C23—C28	−127.2 (3)	C25—C26—C27—C28	1.2 (4)
S3—N3—C23—C24	54.5 (4)	C26—C27—C28—C23	−0.8 (4)

### Hydrogen-bond geometry (Å, º)

**Table:**

*D*—H···*A*	*D*—H	H···*A*	*D*···*A*	*D*—H···*A*
N1—H1···O5^i^	0.86	2.14	2.841 (3)	138
N2—H2*A*···O4^ii^	0.86	2.06	2.876 (3)	158
N3—H3*A*···O10^iii^	0.86	2.18	2.859 (3)	136
N4—H4···O8^iv^	0.86	2.05	2.867 (3)	157
C13—H13···O2	0.93	2.49	3.062 (4)	120
C22—H22*A*···O6^v^	0.96	2.59	3.338 (5)	135
C24—H24···O6	0.93	2.46	3.026 (4)	119

Symmetry codes: (i) −*x*+2, −*y*, −*z*+1; (ii) −*x*+2, −*y*+1, −*z*+1; (iii) −*x*+1, −*y*+1, −*z*+1; (iv) −*x*+1, −*y*, −*z*+1; (v) −*x*+1, −*y*+1, −*z*.
